# High Socioeconomic Status Black Adolescents Attend Worse Schools than Whites

**DOI:** 10.31586/ojer.2025.1160

**Published:** 2025-01-24

**Authors:** Shervin Assari, Hossein Zare

**Affiliations:** 1 Marginalized-Related Diminished Returns (MDRs) Research Center, Los Angeles, CA, USA; 2 Department of Family Medicine, Charles R Drew University of Medicine and Science, Los Angeles, CA, USA; 3 Department of Urban Public Health, Charles R Drew University of Medicine and Science, Los Angeles, CA, USA; 4 Department of Health Policy and Management, Johns Hopkins Bloomberg School of Public Health, Baltimore, MD, USA; 5 School of Business, University of Maryland Global Campus (UMGC), Adelphi, MD, USA

**Keywords:** Family Socioeconomic Status, School Characteristics, Educational Inequality, Minorities’ Diminished Returns, Racial Disparities, School Poverty, Teacher Experience, Academic Outcomes, Structural Barriers, Educational Opportunity

## Abstract

**Background:**

School characteristics — including poverty levels, teacher experience, graduation rates, and college enrollment — are essential determinants of students’ academic outcomes and long-term success. Families often use their socioeconomic resources, such as parental education and household income, to secure access to high-quality schools with favorable attributes. However, Minorities’ Diminished Returns (MDRs) theory suggests that Black families may not experience the same benefits of high family SES due to structural barriers. This study examines the association between family SES and school characteristics, focusing on racial disparities in access to high-quality educational environments.

**Objective:**

To investigate the relationship between family SES (parental education and household income) and multiple school characteristics (poverty, teacher experience, graduation rates, and college enrollment), and to assess racial differences in these associations.

**Methods:**

Data from the Adolescent Brain Cognitive Development (ABCD) study, a national sample of US adolescents, was analyzed. We used multivariate regression models to examine associations between family SES and school characteristics and to test for interactions by race, specifically comparing Black and White adolescents.

**Results:**

Higher family SES was associated with positive school characteristics overall, including lower school poverty, greater teacher experience, and increased graduation and college enrollment rates. However, these positive effects of high family SES on school characteristics were significantly weaker for Black adolescents than for White adolescents. Black adolescents from high-income families were more likely than White adolescents from similar backgrounds to attend schools with higher poverty rates, less experienced teachers, and reduced graduation and college enrollment rates.

**Conclusion:**

Our findings highlight persistent racial inequities in access to educational opportunities, even among families with comparable socioeconomic resources. The diminished returns of family SES for Black adolescents underscore the role of structural barriers in limiting access to high-quality schools. These findings emphasize the need for policy interventions to address systemic inequalities that hinder Black families from fully leveraging their SES to access favorable educational environments.

## 1. Introduction

Educational opportunities — including school poverty levels [1], teacher experience [2], reading performance [3], graduation rates [4,5], and college enrollment [6,7]— play a central role in shaping children’s academic outcomes and future prospects. Areas abundant in high-quality schools typically offer students better academic resources, more experienced teachers, and supportive environments that foster higher graduation rates and post-secondary enrollment [8]. Schools in wealthier, predominantly White suburban neighborhoods tend to have more resources and higher-quality instructional staff, factors that contribute to long-term academic success for their students [9]. In contrast, urban areas, with predominantly Black and Latino schools, commonly face higher poverty rates, fewer experienced teachers, and lower graduation rates, which can limit opportunities for students [10,11].

Families aiming to maximize their children’s educational prospects often prioritize living in areas with high-quality schools [12–15], which are frequently tied to neighborhood affluence, property values, and demographic factors. As a result, families with higher socioeconomic status (SES), often measured through parental education and household income, leverage their economic resources and social capital to secure residence in neighborhoods that offer access to schools with favorable characteristics.

However, for Black families, achieving access to these high-quality educational environments can present greater challenges, even at comparable SES levels to White families [16]. Black families encounter more barriers than their White counterparts, even when they attain similar levels of income and education. This discrepancy is explained by the theory of Minorities’ Diminished Returns (MDRs) [17], which suggests that socioeconomic resources, such as income and education, yield diminished benefits for Black families due to structural racism, segregation, and entrenched economic inequalities. Despite achieving higher SES, Black families are more likely to reside in neighborhoods with fewer resources and schools with higher poverty rates, less experienced teachers, and lower graduation and college enrollment rates than those attended by White students from similar SES backgrounds [16,18]. This phenomenon highlights the persistent racial disparities in educational access, perpetuated by structural inequities that constrain Black families’ ability to convert their socioeconomic resources into educational advantages [19]. Such disparities contribute to sustaining the Black-White achievement gap, even among high-SES children and adolescents [20,21].

The present study examines the association between family SES and a range of school characteristics among a national sample of US adolescents, focusing on racial differences in the alignment between family SES and school quality. We hypothesize that while family income and parental education will generally correlate with more favorable school characteristics (including lower poverty, higher teacher experience, graduation rates, and college enrollment), these associations will be significantly weaker for Black adolescents compared to their White counterparts. In other words, we expect that high-SES Black adolescents will be more likely to attend schools with less favorable characteristics than their White peers of comparable SES. By exploring these disparities, this study seeks to clarify the structural barriers that prevent Black families from fully leveraging their socioeconomic resources within the educational system. Recognizing the extent of these racial inequities can guide policy interventions aimed at dismantling barriers to educational equity.

## 2. Methods

The Adolescent Brain Cognitive Development (ABCD) [22–29] Study is a large-scale, longitudinal study designed to investigate the factors influencing adolescent health and development, including cognitive, emotional, and social outcomes. Using a multistage sampling approach, the ABCD Study recruited participants from diverse regions across the United States, with data collection managed by the ABCD consortium, a collaboration of multiple research institutions. Data collection began in 2016, and participants are followed annually. The study employs extensive assessments across biological, psychological, and social domains to capture the comprehensive influences on adolescent development.

The ABCD sample includes over 11,000 youth, ages 9–10 at baseline, recruited from schools across the United States. This sample was designed to be demographically representative of the U.S. population, with intentional oversampling of specific subgroups to ensure diversity in race, ethnicity, and socioeconomic backgrounds. Informed consent was obtained from both parents and participants, and ongoing data collection includes neuroimaging, cognitive assessments, surveys, and interviews, providing a comprehensive view of each participant’s developmental environment.

For this specific analysis, we included adolescents with complete data on family socioeconomic status (parental education and household income) and relevant school characteristics, including school poverty, teacher experience, graduation rates, and college enrollment. Cases with missing data on these variables were excluded, resulting in a final analytical sample of 10,777 adolescents. The sample included balanced representations of Black and White participants, enabling stratified analyses by race to examine potential disparities.

Data were collected through a combination of self-reported surveys completed by the adolescents and their parents, as well as administrative data related to educational opportunities measured at the neighborhood (residential) level. ABCD data were linked to neighborhood-level data by matching participants’ residential information with contextual variables available in the ABCD residential file. School characteristics, including poverty rates, teacher experience, graduation rates, and college enrollment, were obtained from national databases and cross-referenced with participants’ schools to ensure contextual accuracy. Family socioeconomic status was assessed via parental self-report questionnaires capturing both household income and educational attainment.

Family socioeconomic status (SES) was measured through two indicators. Parental education reflected the highest level of education attained by either parent. Parents were asked, “What is the highest grade or level of school you have completed or the highest degree you have received?” Responses were 0 = Never attended/Kindergarten only; 1 = 1st grade; 2 = 2nd grade; 3 = 3rd grade; 4 = 4th grade 4; 5 = 5th grade; 6 = 6th grade 6; 7 = 7th grade 7; 8 = 8th grade; 9 = 9th grade; 10 = 10th grade 10; 11 = 11th grade; 12 = 12th grade; 13 = High school graduate; 14 = GED or equivalent Diploma; 15 = Some college; 16 = Associate degree: Occupational; 17 = Associate degree: Academic Program; 18 = Bachelor’s degree (ex. BA; 19 = Master’s degree (ex. MA; 20 = Professional School degree (ex. MD; 21 = Doctoral degree. This variable was originally an interval measure with a range between 1 and 21, with a higher score indicating higher educational attainment. This variable was transformed into Jaeger education coding [54] that ranges from 31 to 46. A higher score indicated higher educational attainment. Household income was reported in predefined categories, ranging from less than $25,000 to more than $200,000 annually.

School characteristics in the residential area included several metrics. School poverty level was assessed by the percentage of students eligible for free or reduced-price lunch, used as an indicator of economic disadvantage within schools. Teacher experience was measured as the average years of teaching experience among instructional staff. Reading performance was assessed via average reading proficiency scores of students in the school based on standardized assessments. Graduation rates reflected the percentage of students who successfully graduate within four years, while college enrollment rates indicated the percentage of graduating students who enroll in post-secondary institutions, providing a measure of college preparedness and access. All school characteristics were measured at the residential area level (zip code).

Descriptive statistics were conducted to summarize the demographic and socioeconomic characteristics of the sample. Multivariate regression models were then used to examine associations between family SES indicators (parental education and household income) and each school characteristic (poverty level, teacher experience, graduation rates, and college enrollment). To assess racial disparities in these associations, interaction terms were included in the models to determine whether the effects of family SES on school characteristics varied between Black and White adolescents. Statistical significance was set at p < 0.05. All analyses were conducted using Stata.

The ABCD study received approval from the institutional review boards (IRBs) of each participating institution, ensuring ethical treatment of all participants. For the present analysis, secondary data were utilized under the ABCD’s data-sharing agreement, with all protocols adhering to ethical standards for research involving minors. IRB approval was secured for the use of de-identified ABCD data in this study.

## 3. Results

Table 1 presents descriptive statistics for the sample, which includes 10,777 adolescents. The racial composition of the sample shows that 76.67% (n = 8,263) identified as White, while 23.33% (n = 2,514) identified as Black. Regarding family socioeconomic status, the average parental education level was 42.00 (SE = 0.02), and the mean total family income was 7.31 (SE = 0.02), indicating a relatively high level of SES across the sample. For the residential area school characteristics, the average school poverty level was 48.38% (SE = 0.27), suggesting that nearly half of the students in these areas were eligible for free or reduced-price lunch, an indicator of economic disadvantage within the schools. The mean teacher experience within these schools was 12.66 years (SE = 0.09), reflecting moderate levels of professional experience among instructional staff in the participants’ residential areas. The high school graduation rate within the residential areas was 76.99% (SE = 0.16), indicating that a significant majority of students graduated within four years. Additionally, the average college enrollment rate in these residential areas was 45.28% (SE = 0.10), showing that less than half of the high school graduates enrolled in post-secondary education. These descriptive statistics provide a contextual overview of the demographic and socioeconomic characteristics of the sample and their associated school environments.

Table 2 presents the effects of parental education and total family income on various school characteristics in the residential area, including school poverty, teacher experience, and high school graduation rates.

### 3.1. Residential Area School Poverty

Race had a significant effect on residential area school poverty, with Black race associated with a 13.64-point higher school poverty level (B = 13.64, SE = 0.63, 95% CI [12.41, 14.88], p < 0.001). Parental educational attainment and total family income were both inversely related to school poverty. For each unit increase in parental education, school poverty decreased by 1.84 points (B = −1.84, SE = 0.13, 95% CI [−2.10, −1.58], p < 0.001). Similarly, each increase in total family income was associated with a 3.73-point reduction in school poverty (B = −3.73, SE = 0.13, 95% CI [−3.99, −3.47], p < 0.001).

### 3.2. Residential Area School Teacher Experience

The analysis showed that Black race was associated with a 3.40-point increase in the average years of teaching experience within residential area schools (B = 3.40, SE = 0.24, 95% CI [2.92, 3.88], p < 0.001). Higher parental educational attainment was linked to a small but significant reduction in teacher experience by 0.11 years (B = −0.11, SE = 0.05, 95% CI [−0.21, −0.02], p = 0.024). Total family income also showed a negative association with teacher experience, with each income increase associated with a 0.42-point decrease in teacher experience (B = −0.42, SE = 0.05, 95% CI [−0.53, −0.32], p < 0.001).

### 3.3. Residential Area High School Graduation Rates

The race variable showed that Black adolescents were in areas with a 2.87-point lower high school graduation rate than their White counterparts (B = −2.87, SE = 0.45, 95% CI [−3.75, −1.99], p < 0.001). In contrast, higher parental education was positively associated with graduation rates, resulting in a 0.58-point increase per unit increase in educational attainment (B = 0.58, SE = 0.09, 95% CI [0.39, 0.76], p < 0.001). Total family income was also positively correlated with graduation rates, with a 0.53-point increase associated with each increase in income level (B = 0.53, SE = 0.09, 95% CI [0.34, 0.71], p < 0.001).

### 3.4. Residential Area College Enrollment Rates

Black adolescents were associated with a 1.71-point higher college enrollment rate in their residential area schools compared to White adolescents (B = 1.71, SE = 0.30, 95% CI [1.13, 2.29], p < 0.001). Parental educational attainment also showed a positive association with college enrollment rates, with each unit increase in education linked to a 0.74-point increase in the college enrollment rate in the residential area (B = 0.74, SE = 0.06, 95% CI [0.62, 0.86], p < 0.001). However, total family income did not have a significant effect on college enrollment rates in the residential area schools (B = −0.07, SE = 0.06, 95% CI [−0.19, 0.05], p = 0.255).

Table 3 displays the effects of family socioeconomic status (SES) indicators such as parental education and total family income on various residential area school characteristics, including school poverty, teacher experience, and high school graduation rates.

### 3.5. Residential Area School Poverty

The analysis found that parental education and total family income were significantly associated with lower levels of school poverty in the residential area. Higher parental educational attainment was associated with a 1.78-point reduction in school poverty (B = −1.78, SE = 0.15, 95% CI [−2.07, −1.48], p < 0.001), while each increase in family income was associated with a 4.48-point decrease in school poverty (B = −4.48, SE = 0.16, 95% CI [−4.80, −4.16], p < 0.001). However, race alone did not show a significant association with school poverty (B = −2.58, SE = 12.27, 95% CI [−26.63, 21.47], p = 0.834). Notably, the interaction between race (Black) and total family income was significant, indicating that for Black adolescents, each additional income unit was associated with a weaker reduction in school poverty (B = 2.10, SE = 0.29, 95% CI [1.54, 2.66], p < 0.001), aligning with the concept of Minorities’ Diminished Returns (MDRs).

### 3.6. Residential Area School Teacher Experience

Race showed a significant association with teacher experience, with Black adolescents residing in areas with 12.76 years more teaching experience on average (B = 12.76, SE = 4.74, 95% CI [3.47, 22.04], p = 0.007). However, higher parental education was associated with a small decrease in teacher experience by 0.11 years (B = −0.11, SE = 0.06, 95% CI [−0.23, 0.00], p = 0.045), and each unit increase in family income was similarly associated with a slight reduction in teacher experience (B = −0.18, SE = 0.06, 95% CI [−0.30, −0.06], p = 0.004). The interaction between race (Black) and total family income was also significant, with Black adolescents experiencing an additional decrease in teacher experience for each income unit increase (B = −0.67, SE = 0.11, 95% CI [−0.89, −0.45], p < 0.001).

### 3.7. Residential Area High School Graduation Rates

Higher parental education and family income were both positively associated with high school graduation rates. Each additional unit of parental education was associated with a 0.59-point increase in graduation rates (B = 0.59, SE = 0.11, 95% CI [0.38, 0.80], p < 0.001), while total family income contributed to a 0.70-point increase (B = 0.70, SE = 0.12, 95% CI [0.47, 0.92], p < 0.001). The race variable alone did not show a significant association with graduation rates (B = 5.69, SE = 8.78, 95% CI [−11.53, 22.90], p = 0.517). However, the interaction between race (Black) and total family income was significant, indicating that for Black families, each increase in income was associated with a smaller gain in graduation rates (B = −0.46, SE = 0.20, 95% CI [−0.86, −0.06], p = 0.025), again demonstrating MDRs in educational outcomes.

### 3.8. Residential Area College Enrollment Rates

Race, parental education, and the interaction between race and parental education showed significant associations with college enrollment rates in the residential area. Black adolescents were associated with a 49.04-point higher college enrollment rate in the area (B = 49.04, SE = 5.74, 95% CI [37.80, 60.29], p < 0.001). Parental education was positively associated with a 0.95-point increase in college enrollment for each unit increase (B = 0.95, SE = 0.07, 95% CI [0.82, 1.09], p < 0.001), while total family income did not show a significant effect on college enrollment (B = 0.03, SE = 0.08, 95% CI [−0.11, 0.18], p = 0.648). The interaction between race (Black) and parental education was significant, with Black families experiencing a diminished effect of parental education on college enrollment rates (B = −1.13, SE = 0.15, 95% CI [−1.42, −0.83], p < 0.001).

## 4. Discussion

This study aimed to examine the relationship between family socioeconomic status (SES) indicators — specifically parental education and household income — and various school characteristics in the neighborhoods where families reside. These school characteristics included school poverty levels, teacher experience, graduation rates, and college enrollment. We pursued two primary objectives: first, to assess the overall association between family SES and these school characteristics; and second, to determine whether these associations vary by race, with a focus on disparities between Black and White adolescents. Our study builds on the Minorities’ Diminished Returns (MDRs) framework, which suggests that SES has smaller protective effects for Black families than for White families due to systemic barriers.

The results confirmed our hypotheses. First, we found that higher family SES, as measured by greater parental education and household income, was associated with more favorable school characteristics overall. Adolescents from higher-SES families were more likely to attend schools with lower poverty levels, more experienced teachers, and higher rates of graduation and college enrollment. These findings align with an extensive body of research showing that family SES provides access to educational environments that support academic success and foster post-secondary pathways. Higher-income families generally have the means to move to neighborhoods with robust academic resources, experienced teachers, and supportive school climates, all of which play a critical role in promoting educational achievement.

However, racial disparities emerged in the impact of family SES on school characteristics. For Black adolescents, the benefits of high family SES on neighborhood school characteristics were significantly weaker than those for White adolescents. Despite having high household incomes, Black adolescents were more likely than their White peers from similar SES backgrounds to attend schools with higher poverty rates, less experienced teachers, and lower graduation and college enrollment rates. This disparity is consistent with the theory of Minorities’ Diminished Returns (MDRs) [19], which argues that structural inequalities — including residential segregation, discriminatory housing practices, and exclusionary zoning policies — reduce the advantages of SES for Black families. Even when Black families reach higher SES levels, they are frequently constrained by structural barriers that hinder access to high-quality schools, perpetuating educational disparities and limiting opportunities for advancement [30–35].

These findings extend the growing literature on MDRs by demonstrating that the reduced benefits of family SES for Black families are not limited to individual outcomes [36–42] but also affect the school environments available to their children [43]. Prior research has highlighted that family SES tends to have weaker effects on cognitive, behavioral, and emotional outcomes for Black youth compared to White youth. For example, studies have shown that Black children from higher-SES families do not experience the same cognitive and academic benefits as their White counterparts. Furthermore, Black adolescents with high SES face greater educational and psychological stressors due to structural inequalities embedded in their environments. Our study contributes to this body of work by showing that MDRs also extend to school characteristics, such as poverty levels, teacher experience, and educational outcomes, which are pivotal for youth development and future success.

While this study provides valuable insights, several limitations should be noted. First, our analysis was based on cross-sectional data, which limits our ability to make causal inferences. Future research should employ longitudinal data to assess the long-term effects of school characteristics on academic and developmental trajectories for Black and White adolescents. Additionally, while the ABCD study offers a nationally representative sample, it may not fully capture regional variations in school quality and neighborhood contexts. Future studies could explore other potential mechanisms underlying MDRs, such as perceived discrimination, access to community resources, and school climate, to gain a deeper understanding of the racial disparities in school characteristics.

Future research should prioritize examining the impact of various policies and policy combinations that may mitigate racial disparities in educational access and outcomes. This could include investigating how equitable banking practices, such as expanding credit access and home loan opportunities in majority-minority neighborhoods, influence the ability of Black families to move into higher-quality school districts [44,45]. Additionally, research could explore the effectiveness of enforcing anti-discrimination regulations in housing, school zoning, and educational resource allocation to reduce barriers for Black families in accessing high-quality schools [46]. Studies might also evaluate the impact of increased funding for schools in majority-minority communities, particularly in terms of teacher recruitment, curriculum enhancement, and student support services, to determine whether targeted investments can address educational inequities. By assessing the effectiveness of these policies individually and in combination, future research can provide evidence-based recommendations for dismantling structural barriers and advancing educational equity for marginalized groups [47,48].

The implications of these findings are significant for both educational and public policy. Addressing disparities shaped by MDRs requires more than simply equalizing SES across racial groups [20,21,42,49]. Targeted policy interventions are necessary to dismantle the structural barriers that limit Black families’ access to high-quality schools. Policies that reform zoning laws, expand affordable housing options in affluent neighborhoods, and allocate resources to schools in economically disadvantaged areas are essential for promoting educational equity. Additionally, school- and community-based interventions that provide targeted support for Black students in under-resourced schools could help counterbalance the limitations imposed by structural inequalities, offering these students greater opportunities for academic and social development [50–53].

## Conclusions

Our findings underscore the persistent racial inequities in educational opportunities, even among families with comparable socioeconomic resources. While higher family SES is typically associated with access to schools with favorable characteristics, Black adolescents from high-SES backgrounds continue to face significant barriers that prevent them from fully benefiting from these advantages. Addressing these structural inequities is essential to ensuring that all adolescents, regardless of race, can access high-quality educational environments that foster academic and personal growth. By highlighting the intersection of socioeconomic disparities and structural racism within the educational system, this study reinforces the urgent need for policies aimed at achieving educational equity across racial lines.

## Figures and Tables

**Table 1.** Descriptive Statistics (n = 10,777)

	n	%
Race		
White	8,263	76.67
Black	2,514	23.33
		
Parental Education (Jaeger; 31–46) [[54]]	42.00	0.02
Total Family Income	7.31	0.02
Residential Area School Poverty	48.38	0.27
Residential Area School Teacher Experience	12.66	0.09
Residential Area High School Graduate	76.99	0.16
Residential Area High School Graduate	45.28	0.10

Source: Adolescent Brain Cognitive Development (ABCD)

**Table 2.** Effects of Family Socioeconomic Status on Residential Area Characteristics

	B	SE	95%	CI	p
**Residential Area School Poverty**					
Race (Black)	13.64	0.63	12.41	14.88	<0.001
Parental Educational Attainment	−1.84	0.13	−2.10	−1.58	<0.001
Total Family Income	−3.73	0.13	−3.99	−3.47	<0.001
					
**Residential Area School Teacher Experience**					
Race (Black)	3.40	0.24	2.92	3.88	<0.001
Parental Educational Attainment	−0.11	0.05	−0.21	−0.02	0.024
Total Family Income	−0.42	0.05	−0.53	−0.32	<0.001
					
**Residential Area High School Graduate**					
Race (Black)	−2.87	0.45	−3.75	−1.99	<0.001
Parental Educational Attainment	0.58	0.09	0.39	0.76	<0.001
Total Family Income	0.53	0.09	0.34	0.71	<0.001
					
**Residential Area High School College Enrolment**					
Race (Black)	1.71	0.30	1.13	2.29	<0.001
Parental Educational Attainment	0.74	0.06	0.62	0.86	<0.001
Total Family Income	−0.07	0.06	−0.19	0.05	0.255

Source: Adolescent Brain Cognitive Development (ABCD)

**Table 3.** Effects of Family Socioeconomic Status on Residential Area Characteristics

	B	SE	95%	CI	p
**Residential Area School Poverty**					
Race (Black)	−2.58	12.27	−26.63	21.47	0.834
Parental Educational Attainment	−1.78	0.15	−2.07	−1.48	<0.001
Total Family Income	−4.48	0.16	−4.80	−4.16	<0.001
Race (Black) x Parental Educational Attainment	0.08	0.32	−0.55	0.70	0.813
Race (Black) x Total Family Income	2.10	0.29	1.54	2.66	<0.001
					
**Residential Area School Teacher Experience**					
Race (Black)	12.76	4.74	3.47	22.04	0.007
Parental Educational Attainment	−0.11	0.06	−0.23	0.00	0.045
Total Family Income	−0.18	0.06	−0.30	−0.06	0.004
Race (Black) x Parental Educational Attainment	−0.13	0.12	−0.37	0.12	0.310
Race (Black) x Total Family Income	−0.67	0.11	−0.89	−0.45	<0.001
					
**Residential Area High School Graduate**					
Race (Black)	5.69	8.78	−11.53	22.90	0.517
Parental Educational Attainment	0.59	0.11	0.38	0.80	<0.001
Total Family Income	0.70	0.12	0.47	0.92	<0.001
Race (Black) x Parental Educational Attainment	−0.14	0.23	−0.59	0.31	0.546
Race (Black) x Total Family Income	−0.46	0.20	−0.86	−0.06	0.025
					
**Residential Area High School Graduate**					
Race (Black)	49.04	5.74	37.80	60.29	<0.001
Parental Educational Attainment	0.95	0.07	0.82	1.09	<0.001
Total Family Income	0.03	0.08	−0.11	0.18	0.648
Race (Black) x Parental Educational Attainment	−1.13	0.15	−1.42	−0.83	<0.001
Race (Black) x Total Family Income	−0.12	0.13	−0.38	0.14	0.356

Source: Adolescent Brain Cognitive Development (ABCD)
